# The lumbar erector spinae plane block: a cadaveric study

**DOI:** 10.3906/sag-2107-83

**Published:** 2021-10-30

**Authors:** Serdar KOKAR, Ahmet ERTAŞ, Özlem MERCAN, Fatma Güler YILDIRIM, Ömer Alp TAŞTAN, Kenan AKGÜN

**Affiliations:** 1 Division of Pain Management, Department of Physical Medicine and Rehabilitation, Faculty of Medicine, İstanbul University-Cerrahpasa, İstanbul, Turkey; 2 Department of Anatomy, Faculty of Medicine, İstanbul University-Cerrahpasa, İstanbul, Turkey; 3 Division of Pain Management, Department of Neurology, Faculty of Medicine, İstanbul University-Cerrahpasa, İstanbul, Turkey

**Keywords:** Interventional ultrasonography, regional anesthesia, low back pain

## Abstract

**Background/aim:**

The aim of this cadaveric study was to investigate the erector spinae plane block (ESPB) in lumbar region and to elucidate the possible mechanisms of action of these injections in lumbar radicular pain by means of detecting expected dye dispersion to the neural structures.

**Materials and methods:**

Ultrasound-guided lumbar ESPB was performed in three formaldehyde-embalmed human cadavers. For this purpose, a 10 mL of methylene blue was injected into the fascial space between the L4 transverse process and the erector spinae muscles. Then, the cadavers were dissected, the cephalocaudal and lateral spread of the dye was examined, and the involvement of the dorsal rami, dorsal root ganglia and ventral rami were analyzed. The distribution into the epidural space was also evaluated.

**Results:**

The involvement of the dorsal rami was found to extend up to the T12 level and down to the L5 spinal nerves. Although dye dispersion was detected on the dorsal root ganglion in all specimens, it was found to be limited to one or two levels, unlike the dorsal rami. In half of the specimens, distribution to the ventral ramus and posterior epidural space was observed.

**Conclusion:**

The lumbar ESPB is an interfascial block technique, which can be used to avoid complications, taking advantage of ultrasound. It can be preferred as an alternative to periradicular injection in patients with lumbar radicular pain. It seems to be useful for regional anesthesia, particularly with an increased injectate volume.

## 1. Introduction

Paravertebral block, which is a regional anesthesia technique, has long been used for perioperative analgesia [1]. Recently, the erector spinae plane block (ESPB), an interfascial block technique, has been widely adopted under ultrasound (US) guidance as an alternative method to conventional paravertebral block [2]. The ESPB has been thought to allow local anesthetics to reach the related area without advancing the needle to the paravertebral space, thereby, reducing the risk of complications [3]. Technically, it is a method in which local anesthetics are injected into the fascial space between the erector spinae muscles and transverse process [2].

In a cadaveric study investigating the efficacy of the ESPB technique, Yang et al. [2] demonstrated the involvement of thoracic spinal nerves and the spread to the paravertebral space. Surprisingly, in another cadaveric study, Ivanusic et al. [4] administered methylene blue injection with the US-guided ESPB technique and were unable to reach the dorsal root ganglion (DRG) or the ventral ramus, suggesting that the ESPB was not an alternative to paravertebral block. However, in the literature, there are many case reports in whom ESPB was performed for thoracic surgery [5], breast cancer and reconstructive surgery [6], bariatric surgery [7], rib fractures [8], and postthoracotomy pain syndrome [9].

On the other hand, there is still a limited number of data in the literature regarding the efficacy of ESPB in the lumbar region. Tulgar et al. [10] found that ESPB was an effective method of analgesia in proximal femur and hip surgeries. The authors also reported that a mixture containing 40 mL volume, which they administered at the level of L4 transverse process, was spread around to the neural foramen and spinal nerves. In addition, Alici et al. [11] performed lumbar ESPB in a patient with herpes zoster-induced lower extremity pain and provided long-term analgesia. Furthermore, several cases have been described in patients diagnosed with radicular pain due to lumbar disc herniation and administered lumbar ESPB [12,13]. Harbell et al. [14] reported that a lumbar ESPB injection had limited craniocaudal spread compared to injection into the thoracic region without spread to the DRG, ventral rami, or paravertebral space. In another cadaveric study, however, the injected solution reached and passed the anterior of the transverse process and, even, the ventral rami were stained in some of the samples [15]. Both studies showed that dorsal rami were stained in all procedures. However, many authors have emphasized that further cadaveric studies are needed to elucidate the mechanism of action of these injections.

Besides many pharmacological and nonpharmacologic approaches, epidural steroid and local anesthetic injections are the treatment options, which are frequently used in the treatment of low back pain, particularly in patients with radicular complaints [16,17]. These procedures include interlaminar, transforaminal, and caudal approaches [18]. However, each poses certain risks of complications such as dural puncture, epidural abscess, nerve injury, and paralysis [18,19]. Therefore, it is likely to avoid a considerable amount of these complications performing interfascial blocks. Furthermore, in patients with altered lumbar spine anatomy, this challenge can be overcome by permorfing fascial blocks.

In the present study, we aimed to investigate the ESPB in lumbar region and to elucidate the possible mechanisms of action of these injections in lumbar radicular pain by means of detecting expected dye dispersion to the neural structures. The study provides an insight into the conflicting results of two previous studies [14,15] and a significant contribution to the literature.

## 2. Materials and methods

### 2.1. Study design

The present human cadaveric study was approved by the institutional Ethics Committee (Date: 04.03.2020, No: 37451). All cadavers used in the study were legally donated to the Department of Anatomy. In this study, US-guided lumbar ESPB was administered to three adult formaldehyde-fixed cadavers. Those aged below 50 years and having a previous lumbar surgery were excluded from the study. Unilateral injection was administered in two of them, while bilateral block was performed in the other one to evaluate possible variability of the results in different cadavers and both sides of the same cadaver.

### 2.2. Interventional procedure

**Figure 1:**
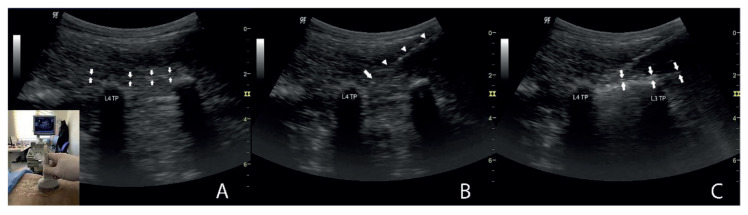
(A) Fascial space (arrows). The probe in the longitudinal parasagittal orientation (left bottom). (B) The spinal needle advancing from the cephalic to caudal direction using in-plane technique (arrow heads). The needle tip (arrow) located in the target fascial space. (C) Linear spread of the fluid throughout the fascial space (arrows). L3 TP, L3 transverse process; L4 TP, L4 transverse process.

All cadavers were placed in the prone position. All injections were performed by a single pain specialist having more than 15 years of experience in US-guided interventional procedures. A low-frequency (2 to 5 MHz) curvilinear transducer (LOGIQ P5, GE Healthcare, Buckinghamshire, UK) was used to visualize deep structures. At first, an US scan was performed to identify the L4 spinal level. By sliding the probe laterally in a longitudinal parasagittal orientation, the L4 transverse process together with the L3 and/or L5 was, then, visualized (Figure 1A). After imaging the erector spinae muscles lying above the transverse processes, a 90-mm, 20-gauge Quincke-type spinal needle was advanced toward the target in the craniocaudal direction using the in-plane technique. Deep to the erector spinae muscle, no advance of the needle was attempted, when it reached the interfascial plane between the muscle group and the transverse process of L4 (Figure 1B). Primarily, the linear spread of the fluid was observed by injecting 2 mL of 0.9% saline solution, and it was confirmed to be in the fascial space (Figure 1C). Subsequently, 10 mL of methylene blue was injected, and the intervention was terminated, after recording the direction of the spread.

### 2.3. Dissection

Dissections were performed by the anatomists within one hour after the injections. In the first step, the skin and subcutaneous tissues were removed. Subsequently, the latissimus dorsi and erector spinae muscles were identified and removed. The stained areas on the anterior and posterior parts of the erector spinae muscles were detected, and the intertransversarii and psoas major muscles were examined. It was searched for which of the dorsal rami of the spinal nerves among the erector spinae muscle fibers were stained with methylene blue. These nerves were followed until the intervertebral foramen. The vertebral laminae and articular processes were dissected, and then whether the DRGs and ventral rami were stained was evaluated. Spinous processes were also dissected, and the distribution of dye in the epidural space was assessed. Finally, the cauda equina and medulla spinalis were excised to search the dye in the anterior epidural space and posterior to the intervertebral discs. At each stage of the dissection, the extent of lateral and cephalocaudal distribution was noted.

## 3. Results

**Figure 2:**
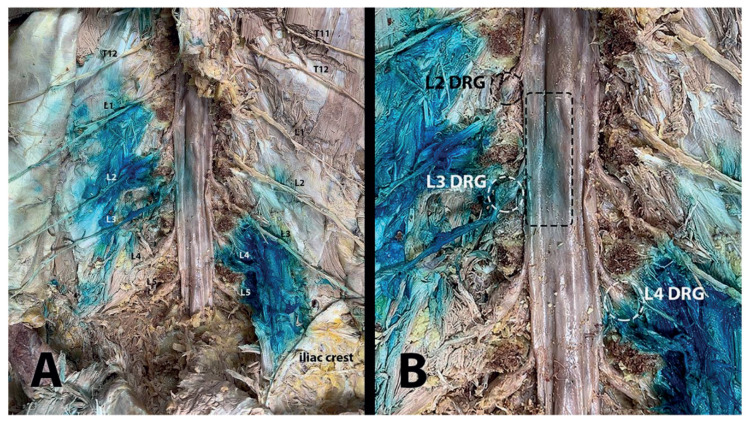
(A) Involvement of posterior rami. Please note that the spread of methylene blue is mostly unidirectionally with predominantly cephalic or caudal staining (each posterior ramus written in the figure). Lateral spread extends up to medial part of iliac crest. (B) Staining of dorsal root ganglia (dashed circles) and epidural region (dashed rectangle).

The whole injection and dissection procedures were performed successfully, and all three cadavers were included in the study. Through the superficial to the deep layers based on a step-by-step approach, there was no significant dye within the skin or subcutaneous layers. However, a nonsignificant amount of methylene blue was noticed to have leaked along the needle path in two cadavers. In all injections, the dye was spread along both the superficial and deep fasciae of erector spinae muscles, mainly in the deep plane. The spread in the lateral direction was observed to have reached the medial part of the iliac crest (Figure 2A). In addition, in all specimens, staining was detected in variable amounts on transversospinales (multifidus, rotatores), intertransversarii, quadratus lumborum, and psoas major that are deep muscles of the vertebral column.

**Figure 3:**
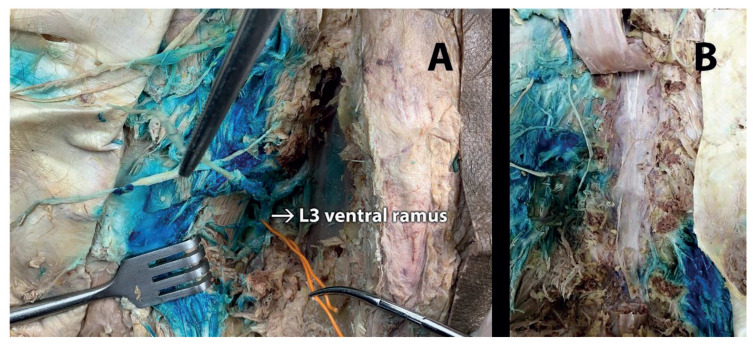
(A) Staining of ventral ramus of L3 hold by an orange wire. (B) No staining in anterior epidural space or posterior to the intervertebral discs.

The involvement of dorsal rami was found to extend up to the T12 level and down to the L5 spinal nerves (Figure 2A). These levels differed among the samples (Table 1). Although dye dispersion was detected on the DRG in all specimens, it was found to be limited to one or two levels, unlike the dorsal rami (Table 2, Figure 2B). Staining was observed in the ventral ramus in half of the samples; however, it was limited to a single (L3) level (Figure 3A). The stained dorsal rami, DRG, and ventral rami conformed to the spread direction as visualized sonographically. The distributions mostly occurred unidirectionally (cephalic or caudal).

In addition, the methylene blue was observed in the posterior epidural space in half of the samples, corresponding to the L3-L4 levels (Figure 2B). No dye was observed in the anterior epidural space or posterior to the intervertebral discs (Figure 3B).

Furthermore, there were variations in distribution of both sides of the same cadaver and among the cadavers.

## 4. Discussion

The erector spinae muscles extend longitudinally along the vertebral column, which facilitates the craniocaudal spread in ESPB [20]. Besides, through the gap created by fasciae, lateral spread can be achieved, extending to the medial part of the iliac crest, i.e. the attachment site of iliocostalis lumborum which constitutes the lateral part of the erector spinae muscles [4,21]. In the present study, we investigated the ESPB in lumbar region in cadaveric specimens. We administered the injection into the space between the deep fascia of the erector spinae muscles and transverse processes. We observed that the methylene blue was spread mainly along the deep and medio-lateral plane. However, since the fascial space is connected to the superficial fascia of the erector spinae muscles [2], staining also occurred on the posterior surface of the muscles. In this context, we do not agree with the opinion of Harbell et al. [14] that thoracolumbar fascia (TLF) restricts the posterior spread. Although TLF is thicker in the lumbar region as stated, it is unlikely for it to be a sufficient barrier to prevent the spread of the injection solution to the posterior, and the solution can find its way to spread posteriorly.

**Figure 4:**
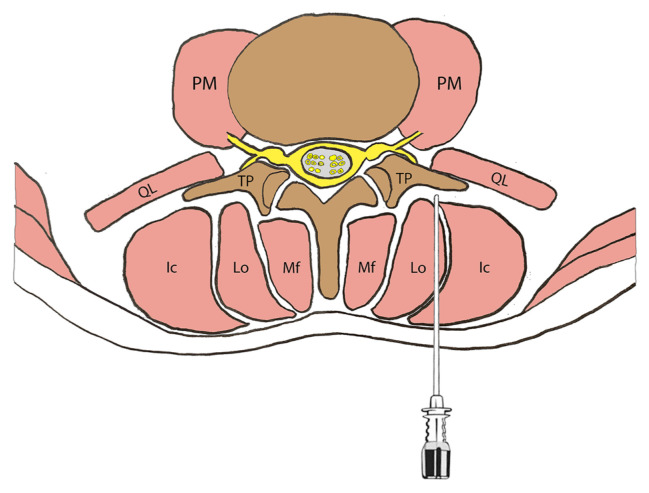
A schematic view of lumbar vertebrae and related muscles. Deep to the erector spinae muscles, the spinal needle extending into the fascial space (target) between the transverse process and muscle group. PM, Psoas Major; QL, Quadratus Lumborum; Ic, Iliocostalis; Lo, Longissimus; Mf, Multifidus; TP, transverse process.

The access of the injectate to the anterior aspect lies over and below the transverse process (Figure 4). The deep muscles of the vertebral column (i.e., psoas major, quadratus lumborum, and intertransversarii) limit the distribution to the anterior to some extent [22]. Therefore, the ventral rami may have been involved just in half of the injections in our study. In a study, de Lara González et al. [15] reported dissemination before the transverse process in most of the samples (75%), but the spread to the ventral rami was limited (17%).

Although DRG involvement was identified in all injections, it might have been limited to one or two levels due to the same reason. On the other hand, when the ESPB is performed in the lateral decubitus position, whether the distribution can be restricted in the lateral direction and head toward the DRG/epidural space located medially may be the subject of another study. However, based on the staining of the DRG and dorsal rami in all specimens, we consider that lumbar ESPB is likely to share the similar mechanism of action as the periradicular injection. In this regard, our results are not consistent with the cadaveric study findings of Harbell et al. [14]. The aforementioned authors reported no spread to the DRG; however, in all cadavers, DRG was stained in our study, which is probably the most important finding of the present study. This discrepancy between the studies can be attributed to the needle positioning over the medial or lateral part of the transverse process and anatomical differences. Of note, in the study of Harbell et al. [14], no anterior spread was observed in any of nine injections, which seems to contradict with many case reports [12,13,23]. The most important step of ESPB is to position the needle tip exactly in the fascial space. It should be kept in mind that the spread may remain limited if the needle tip is positioned within the muscles over the transverse process.

In a cadaveric study, Adhikary et al. [24] reported epidural distribution in their samples in which they administered thoracic ESPB with 20 mL of a radiocontrast dye mixture. In another study using a 20-mL volume, Harbell et al. [14] reported no lumbar epidural spread. In the current study, we used 10 mL of volume. There was no epidural spread for each injection. However, in half of the injections, the dye reached the dura mater. It is likely that, by increasing the volume, to eliminate this limitation and even to reach the anterior epidural space. In this context, it should be kept in mind that the objective of the present study was to elucidate the possible efficacy of these injections, if administered in patients with lumbar radicular pain. It is not appropriate to use steroids, one of the main components of these injections, in such high volumes as the methylene blue used in the current cadaveric study. In routine clinical practice, we usually use 2 to 4 mL of dexamethasone for lumbar epidural injections. In this case, how much of the diluted steroid in the 20 mL mixture arrives at the target would be another question to be answered. Considering all these reasons, it cannot be speculated that lumbar ESPB is an exact alternative to epidural injections.

Nonetheless, the significance of the aforementioned issues is still controversial regarding the interventional procedures in patients with lumbar radicular pain. Although review of the literature reveals more favorable results for epidural injections [25,26], some authors have suggested that epidural injections are not superior to selective nerve blocks [27,28]. We believe that further studies are needed to elucidate these discrepancies and to gain a better understanding of this topic.

On the other hand, the present study has some limitations due to the nature of cadaveric studies. Cadaveric models may not represent living subjects exactly. A more dynamic and extensive distribution of the injections is supposed to occur in living tissues than cadavers [20]. This difference may result from tissue tension and pressure alterations [4]. Muscle contraction in livings induces not only bone movement but also fascial stretching. Thus, the local anesthetic, which has already moved on passively along the fasciae, is transported similarly to a pump mechanism [29]. In addition, the distribution can be affected by the use of formaldehyde-embalmed cadavers, instead of fresh cadavers. According to this theoretical rationale, the factors that we consider limitations to this study support our findings implicitly.

Another limitation is related to the spread direction of methylene blue. Ivanusic et al. [4] found cephalic distribution in their cadaveric study which thoracic ESPB was performed. In another cadaveric study in which lumbar ESPB was performed, de Lara González et al. [15] reported that the spread could occur in both directions. Schoenfeldt et al. [30] reported that their clinical observations in these procedures were in favor of the caudal spread. The authors also emphasized that the conflict could be explained by the difference between cadavers and living subjects. In our study, we administered all of the injections between the L4 transverse process and the deep fascia of the erector spinae muscles. The fluid was spread predominantly in the cranial direction in half of the injections, whereas caudal spread was prominent in the other half. Consequently, T12-L3 dorsal rami and L2, L3 DRG were stained with cranial spread, while L3–L5 dorsal rami and L4, L5 DRG were involved in caudal spread (Tables 1 and 2). The lack of any staining in the S1 dorsal rami and limited spread in the caudal direction in our study are consistent with previous findings of the studies of Harbell et al. [14] and de Lara González et al. [15]. Although it seems to complicate performing the block at the targeted level, it was considered a condition, which could be avoided. Therefore, we administered 2 mL of saline solution (0.9%) into the fascial space before injecting the methylene blue. Thus, we confirmed that the needle was in the proper location and documented the direction of the fluid spread. The results after dissection proved that the stained spinal nerves and DRG coincided with the direction of distribution as evidenced by US imaging. In the light of these findings, we recommend injecting a saline solution to the target point and identifying the direction of the spread in the treatment of low back pain. If no spread occurs in the intended direction, it would be wise to terminate the procedure by drawing the needle back and to perform the procedure at another level. However, since regional anesthesia requires higher volumes with a greater extent of spread [10], drawing the needle back and identifying the direction of the spread as in the lumbar radicular pain may be clinically less significant in these procedures.

The guidance of the US has certain merits, including having no radiation exposure, being relatively inexpensive and accessible thanks to its portable design [31]. All of those are the distinctive features of the US from other imagining technologies. In addition, it can visualize soft tissues and vessels through real-time visualization [31,32].

## 5. Conclusion

Based on our study results, we suggest that the ESPB injection using 10 mL of volume may be an alternative to periradicular injection. If higher volumes are administered, these blocks may be preferred to epidural anesthesia. In addition, the ESPB reduces the complication risks owing to being a superficial injection technique. Therefore, the lumbar ESPB is an option, which should be kept in mind, particularly in case of altered lumbar spine anatomy (i.e. lumbar spondylosis, spinal stenosis, scoliosis, or fractures), bleeding diathesis, and allergy to contrast materials. Additionally, it may be considered the first-line treatment or anesthesia method taking the advantages of US. The level of recommendations presented is inevitably confined to further clinical researches in this field.

## Figures and Tables

**Table 1:** Staining of posterior rami†.

Level	Cadaver no. 1		Cadaver no. 2 (cephalic)	Cadaver no. 3 (caudal)
	Left (cephalic)	Right (caudal)		
T12				
L1				
L2				
L3				
L4				
L5				

Blue bars represent the level of involvement.

† Methylene blue was used for staining.

**Table 2:** Staining of dorsal root ganglia†.

Level	Cadaver no. 1		Cadaver no. 2 (cephalic)	Cadaver no. 3 (caudal)
	Left (cephalic)	Right (caudal)		
L2				
L3				
L4				
L5				

Blue bars represent the level of involvement.

† Methylene blue was used for staining.

## References

[1] Karmakar MK Thoracic paravertebral block Anesthesiology 2001 95 3 771 780 10.1097/00000542-200109000-00033 11575553

[2] Yang HM Choi YJ Kwon HJ OJ Cho TH Comparison of injectate spread and nerve involvement between retrolaminar and erector spinae plane blocks in the thoracic region: a cadaveric study Anaesthesia 2018 73 10 1244 1250 10.1111/anae.14408 30113699

[3] Onishi E Toda N Kameyama Y Yamauchi M Comparison of Clinical Efficacy and Anatomical Investigation between Retrolaminar Block and Erector Spinae Plane Block BioMed Research International 2019 2578396 10.1155/2019/2578396 31032339 PMC6458933

[4] Ivanusic J Konishi Y Barrington MJ A Cadaveric Study Investigating the Mechanism of Action of Erector Spinae Blockade Regional Anesthesia and Pain Medicine 2018 43 6 567 571 10.1097/AAP.0000000000000789 29746445

[5] Forero M Rajarathinam M Adhikary S Chin KJ Continuous Erector Spinae Plane Block for Rescue Analgesia in Thoracotomy After Epidural Failure: A Case Report A & A Case Reports 2017 8 10 254 256 10.1213/XAA.0000000000000478 28252539

[6] Ohgoshi Y Ikeda T Kurahashi K Continuous erector spinae plane block provides effective perioperative analgesia for breast reconstruction using tissue expanders: A report of two cases Journal of Clinical Anesthesia 2018 44 1 2 10.1016/j.jclinane.2017.10.007 29065334

[7] Chin KJ Malhas L Perlas A The Erector Spinae Plane Block Provides Visceral Abdominal Analgesia in Bariatric Surgery: A Report of 3 Cases Regional Anesthesia and Pain Medicine 2017 42 3 372 376 10.1097/AAP.0000000000000581 28272292

[8] Hamilton DL Manickam B Erector spinae plane block for pain relief in rib fractures British Journal of Anaesthesia 2017 118 3 474 475 10.1093/bja/aex013 28203765

[9] Forero M Rajarathinam M Adhikary S Chin KJ Erector spinae plane (ESP) block in the management of post thoracotomy pain syndrome: A case series Scandinavian Journal of Pain 2017 17 325 329 10.1016/j.sjpain.2017.08.013 28919152

[10] Tulgar S Selvi O Senturk O Ermis MN Cubuk R Clinical experiences of ultrasound-guided lumbar erector spinae plane block for hip joint and proximal femur surgeries Journal of Clinical Anesthesia 2018 47 5 6 10.1016/j.jclinane.2018.02.014 29522966

[11] Alici HA Ahiskalioglu A Aydin ME Ahiskalioglu EO Celik M High volume single injection lumbar erector spinae plane block provides effective analgesia for lower extremity herpes zoster Journal of Clinical Anesthesia 2019 54 136 137 10.1016/j.jclinane.2018.11.009 30502675

[12] Celik M Tulgar S Ahiskalioglu A Alper F Is high volume lumbar erector spinae plane block an alternative to transforaminal epidural injection? Evaluation with MRI. Regional Anesthesia and Pain Medicine 2019 rapm-2019-100514 10.1136/rapm-2019-100514 30992410

[13] Karaca O Pinar HU Is high dose lumbar erector spinae plane block safe? Journal of Clinical Anesthesia 2020 62 109721 10.1016/j.jclinane.2020.109721 31955130

[14] Harbell MW Seamans DP Koyyalamudi V Kraus MB Craner RC Evaluating the extent of lumbar erector spinae plane block: an anatomical study Regional Anesthesia and Pain Medicine 2020 45 8 640 644 10.1136/rapm-2020-101523 32546551

[15] De Lara González SJ Pomés J Prats-Galino A Gracia J Martinez-Camacho A Anatomical description of anaesthetic spread after deep erector spinae block at L-4 Revista Espanola de Anestesiologia y Reanimacion 2019 66 8 409 416 10.1016/j.redar.2019.07.001 31488244

[16] Manchikanti L Benyamin RM Falco FJ Kaye AD Hirsch JA Do Epidural Injections Provide Short- and Long-term Relief for Lumbar Disc Herniation? A Systematic Review Clinical Orthopaedics and Related Research 2015 473 6 1940 1956 10.1007/s11999-014-3490-4 24515404 PMC4419020

[17] Vlaeyen JWS Maher CG Wiech K Van Zundert J Meloto CB Low back pain Nature Reviews Disease Primers 2018 4 1 52 10.1038/s41572-018-0052-1 30546064

[18] Bicket MC Chakravarthy K Chang D Cohen SP Epidural steroid injections: an updated review on recent trends in safety and complications Pain Management 2015 5 2 129 146 10.2217/pmt.14.53 25806907

[19] Manchikanti L Malla Y Wargo BW Cash KA Pampati V A prospective evaluation of complications of 10,000 fluoroscopically directed epidural injections Pain Physician 2012 15 2 131 140 10.36076/ppj.2012/15/131 22430650

[20] Forero M Adhikary SD Lopez H Tsui C Chin KJ The Erector Spinae Plane Block: A Novel Analgesic Technique in Thoracic Neuropathic Pain Regional Anesthesia and Pain Medicine 2016 41 5 621 627 10.1097/AAP.0000000000000451 27501016

[21] Kalimo H Rantanen J Viljanen T Einola S Lumbar muscles: structure and function Annals of Medicine 1989 21 5 353 359 10.3109/07853898909149220 2532525

[22] de Araújo Baptista VI Mayer WP Eustáquio da Silva R de Vasconcellos Fontes RB da Silva Baptista J An alternative didactic, functional and topographic systematization of the spinal muscles Annals of Anatomy - Anatomischer Anzeiger: Official Organ of the Anatomische Gesselschaft 2017 213 47 51 10.1016/j.aanat.2017.05.006 28602826

[23] Brandão J Graça R Sá M Cardoso JM Caramelo S Lumbar erector spinae plane block: Successful control of acute pain after lumbar spine surgery - A clinical report Revista Espanola de Anestesiologia y Reanimacion 2019 66 3 167 171 10.1016/j.redar.2018.10.005 30522818

[24] Adhikary SD Bernard S Lopez H Chin KJ Erector Spinae Plane Block Versus Retrolaminar Block: A Magnetic Resonance Imaging and Anatomical Study Regional Anesthesia and Pain Medicine 2018 43 7 756 762 10.1097/AAP.0000000000000798 29794943

[25] Singh S Kumar S Chahal G Verma R Selective nerve root blocks vs. caudal epidural injection for single level prolapsed lumbar intervertebral disc - A prospective randomized study Journal of Clinical Orthopaedics and Trauma 2017 8 2 142 147 10.1016/j.jcot.2016.02.001 28720990 PMC5498739

[26] Van Boxem K Cheng J Patijn J Van Kleef M Lataster A 11. Lumbosacral radicular pain Pain Practice: The Official Journal of World Institute of Pain 2010 10 4 339 358 10.1111/j.1533-2500.2010.00370.x 20492580

[27] Narozny M Zanetti M Boos N Therapeutic efficacy of selective nerve root blocks in the treatment of lumbar radicular leg pain Swiss Medical Weekly 2001 131 5–6 75 80 10.4414/SMW.2001.09689 11383229

[28] Park KD Lee WY Nam SH Kim M Park Y Ultrasound-guided selective nerve root block versus fluoroscopy-guided interlaminar epidural block for the treatment of radicular pain in the lower cervical spine: a retrospective comparative study Journal of Ultrasound 2019 22 2 167 177 10.1007/s40477-018-0344-z 30519991 PMC6531573

[29] Elsharkawy H Pawa A Mariano ER Interfascial Plane Blocks: Back to Basics Regional Anesthesia and Pain Medicine 2018 4 43 341 346 10.1097/AAP.0000000000000750 29561295

[30] Schoenfeldt J Guffey R Fingerman M Cadaveric study investigating the mechanism of action of erector spinae blockade Regional Anesthesia and Pain Medicine 2019 44 280 10.1136/rapm-2018-100190 30635502

[31] Provenzano DA Narouze S Sonographically guided lumbar spine procedures Journal of Ultrasound in Medicine: Official Journal of the American Institute of Ultrasound in Medicine 2013 32 7 1109 1116 10.7863/ultra.32.7.1109 23804333

[32] Narouze SN Atlas of ultrasound-guided procedures in interventional pain management 2nd ed New York, USA Springer 2018

